# Convulsions in Children

**Published:** 1885-06-20

**Authors:** Arch. Dixon

**Affiliations:** Kentucky


### Southern Medical Record.

EDITORS:

THOMAS S. POWELL, M.D. R. C. WORD, M.D.

*R. C. WORD, M.D., Managing Editor.*

☞All Communications and Letters on Business connected with the RECORD must be addressed to the Managing Editor.

VOL. XV. ATLANTA, GA., JUNE 20, 1885. No. 6.

### ORIGINAL AND SELECTED ARTICLES.

### CONVULSIONS IN CHILDREN.

By ARCH. DIXON, M.D., of Ky.

Spasms in children is a subject of far more interest to the general practitioner than is usually accorded it; especially is the question of the causation of convulsions one of absorbing interest, and resolves itself into two distinct departments, one of which is strictly clinical in character, whilst the other is more strictly physiological. It is one thing for the medical man to ascertain what are the particular individual conditions, physical, pathological, or otherwise, which have contributed to induce an attack of convulsions (to ascertain which he studied the *predisposing* and *exciting* causes of the disease), but it is quite a different problem, and far more difficult, when he endeavors to unravel, by anatomico-physiological data, the actual mode of production of the convulsions. In this latter part of the inquiry he has to do with what are called "proximate" causes and is brought face to face with a problem so intricate and abstruse, that it is still involved in great obscurity, and concerning which the most opposite views are held by leading pathologists and physiologists; and in the very outset the great question of the relation of nerve to muscle confronts us. It cannot be denied that the activity of contractile protoplasm is in no way essentially dependent on the presence of nervous elements. It was Haller who first laid the foundation of our knowledge of physiology of muscle and nerve by establishing the doctrine of muscular and nervous irritability, the ground work of reflex action. The large majority of authors look upon convulsions in children, save those dependent upon cerebal lesions, as reflex in character and due to some irritation which produces these exagerated reflex phenomena. All the machinery a reflex action demands is a sentient surface, (external or internal) connected by an afferent nerve with a central nerve cell or group of connected nerve cells, which is in relation by means of a motor or efferent nerve, or nerves with a muscle, or muscles, or some other irritable tissue elements, capable of responding, by some change in their condition, to the advent of efferent impulses. Saltman attributes the great tendency to reflex phenomena in young animals until the tenth day to the absence of reflex inhibitory centres in the brain and spinal cord. We might infer from this that the increase of reflex action sufficient to produce general or partial convulsions was due to a general or partial paralyzation of the inhibirtory reflex centres in the brain and spinal cord, and that there are such inhibitory centres cannot be denied. We, ourselves, are conscious of being able by an effort of the will to stop reflex movements, such, for instance, as are induced by tickling. There must, therefore, be in the brain some mechanism or other for preventing the normal development of the spinal reflex actions. Setschenow has demonstrated, not only by experiments upon frogs, but upon mammals, that such an inhibitory centre does exist in the brain, and that, as the governor upon an engine, controls, guides and restrains reflex phenomena, which, owing to the inherent contractility and irritability of muscular and nervous tissue, but for this controlling and restraing influence would keep these tissues in a general convulsive state. All day long and every day multitudinous afferent impulses from eye and ear and skin, and muscle, and other tissues and organs are streaming into our nervous system ; and did each afferent impulse issue as its correlative efferent motor impulse our life would be a prolonged convulsion. As it is, by the checks of cerebral and spinal activities induced by this grand inhibitory centre in the brain, aided by the lesser inhibitory centres in the cord, all these impulses are drilled, marshalled, and kept in hand in orderly array till a movement is called for ; and thus we are able to execute at will the more complex bodily maneuvers, knowing only why, and unconscious, or but dimly conscious *how* we carry them out.

Let us go back and ask what is the relation of nerve to muscle? Is it the medium through which stimulus is applied to muscle in order to produce contractions ? or is it, as some have contended, the restaining an inhibitory source of that power which prevents undue contraction of muscle, to which it is so prone, by reason of its inherent contractility and irritability ?

It has already been asserted, and abundant evidence can be brought to bear in proof, that the activity of contractile protoplasm is in no way dependent on the presence of nervous elements. A very pretty and plausible argument can be, and has been *made*, with this assertion as the ground work, that inherent muscular contractility and irritability is kept in hand, controled, guided and governed by this vast net work of nerves, which penetrate and are interwoven with almost every tissue in the human body, but not in the manner usually accepted and taught by physiologists. The stimulating power of the nerves is denied, and the contrary is asserted, that reflex action is not due to an impulse transmitted by an afferent nerve to a cell or group of nerve cells in relation to a muscle, by means of a motor, or efferent nerve, and that this impulse is further transmitted by this efferent nerve causing contraction of the muscle by stimulation of the end plates ; but that this impulse, caused by irritation in some form, produces a paralytic condition of the nerve tissues, which controls muscular contractility and the muscles being freed from this inhibitory power, at once contracts.

It is claimed further, that, by irritant impulses transmitted to the inhibitory centre of Setschenow paralysis of this grand grand inhibitory centre may be, and is produced, and as a consequence, general convulsions ensue. Be this as it may, it is beyond the province of this paper to enter very deeply into a discussion of the actual mode of production of convulsions, which, as I have before remarked, is a question about which the views of leading pathologists and physiologists are widely divergent. We must, therefore, rely rather upon our own observations, which daily confirms the great liability of children to reflex spasms. It is useless to describe to you the symptomatology of convulsions, the rolling of the eyes upward, the twitching of the facial muscles, the closure of the jaws, the tetanic stiffness of the limbs, interrupted by short twitchings, the gradual subsidence of these phenomena, leaving the little patient in a comatose condition, before recovering from which the attack may be renewed, recurring again and again before consciousness is restored. In these attacks reflex action is occasionally preserved, but in most cases, it is entirely absent.

The duration of the paroxysm is of great importance. The convulsions, interrupted by short intervals of coma, may continue for hours, and the interference with respiration, the venous stasis in the brain, and the exhaustion of the vital energies, may finally cause death. But recovery may take place, although the convulsions have lasted many hours or even for days. The common form of convulsions, lasting a few minutes, has usually subsided when the physician arrives. The child is then usually found in a comatose condition, which passes imperceptibly into a sound sleep that may last for hours, and from which the child awakes as if nothing had happened. When called to a case of this kind, and finding the child in convulsions, no time is to be lost in seeking for the cause ; treatment must be immediately begun.

In the first place, it is well for one's own comfort, as well as for that of the family, to remember that children do not usually die in convulsions, and a statement of this sort reassures and comforts the alarmed parents. This may be remarked whilst you are getting your remedies ready for the relief of your patient. And I know of no remedy which fills the indications more promptly or more certainly than chloroform ; in fact, I never waste time with other remedies, but immediately begin its use ; after a few respirations the spasmodic irritation subsides and the inhalations may be continued until the complete cessation of the convulsions. As a matter of course the pulse and respirations must be carefully watched during the administration. I have never observed any unpleasant effects, although I have given it in numerous cases, and have even gone so far as to entrust the attendants with the administration of it, and thus far have had no reason to regret it. The contra indications to its use are rare, and I shall not hesitate to give it when there was a cyanotic condition of the face, nor where convulsions have developed during the course of a bronchitis, or a broncho-pneumonia. It should not be employed if the child is already in a condition of collapse, with a very small and rapid pulse, and cool extremities.

Another remedy I have employed with excellent results is morphia hyperdermically ; and in the absence of chloroform, I do not hesitate to make use of it; and occasionally in cases which the chloroform only mitigates the spasm without relieving it, the morphia has the most happy effect.

It is also an excellent plan to order a warm bath to be got ready. Fulness or weakness of the pulse will be an aid to the diagnosis, as will also the condition of the head, if it be hot or cold ; if the fontanelle be tense and protruding, or sunk and retracted, and if the face be flushed or pale. Having stripped the child, observe if the thighs are flexed upon the belly; if so, and the head be hot and the fontanelle throbbing and prominent, there is congestion, which may be the cause or the result of the convulsions. But in either case it is good treatment to put your child in the hot bath, into which has been thrown a little mustard, at the same time making a cold application to the head. The same treatment holds good if the head be cold or the fontanelle depressed; and then employ friction to arouse the action of the skin, at the same time a little ammonia may be held to the nose and a few drops of whiskey in a little water to moisten the lips. As soon as the convulsions have ceased the cause of the disease must be taken into consideration.

In the first place, it is important to know whether the convulsions are due to a material lesion of the brain. If the spasms are unilateral in character and upon their recurrence remain restricted to the same side of the body, the inference is that they are cerebal in origin ; but it should not be forgotten that bilateral convulsions may occur with a unilateral lesion of the brain, and on the other hand that unilateral spasms are occasionally observed, although no cerebral affection be present. It will be well to remember the predisposition of rachitic children to spasms. Occasionally the cutting of a tooth, under especially unfavorable circumstances, may produce convulsions, but these cases are rare, and other exciting causes should be looked for. Among these, irritative conditions of the organs undoubtedly occupy the first place. Convulsive attacks are not infrequent in the dyspeptic conditions of infants, and those cases in which eclampsia occurs soon after emotional excitement of the mother, also belong to this category, as they can only be explained by an injurious influence of the milk on the digestive organs of the child. But overloading of the stomach or intestinal canal may cause severe convulsions in later childhood, and these cases indicate the plan of treatment by emetics and purgatives, which rapidly remove the offending material. A few drops of squibbs, fl. extract of ipecac, followed by a grain of calomel and sugar is usually all that is required ; occasionally it may be necessary to give an enema, especially if the bowel be much distended. A little warm water and soap answers well. In those cases in which worms are known to be present, it is advisable to add a grain or two of santonine to your calomel. It may be possible that worms do sometimes cause convulsions, but I have never seen a case in which they could with certainty be attributed to this cause. In eclampsia preceded and followed by fever—and this may be the case in dyspeptic convulsions—it is important to examine other organs, acute affections of which begin oftentimes in children with fever and severe convulsions, as for example, primary pneumonia, pleurisy and enteritis. The diagnosis of pneumonia beginning this way, may be different at first, because examination of the chest reveals nothing abnormal at this early stage, and it may be doubtful for a few days whether we do not have to deal with an acute inflammatory affection of the brain. The cause of the convulsions in these cases is not very clear. They might be attributted either to reflex irritation starting in the lungs, pleura or intestines, or to the high fever, which is sufficient to produce convulsions in irritable children.

The convulsions which occasionally occur in the initial stage of acute infectious diseases, (scarlatina, measles, smallpox, etc.), probably belong in this febrile category, though, doubtless, the infectious material in the blood aids largely in their production. In all such cases the treatment can only be given symptomatically, by the appliation of cold to the head, graduated baths, enemas and mild purgatives, and by the administration of nerve sedatives, at the head of which stands chloral hydr. and the bromides. Uræmia and intermittent fever may also be classed among the acute diseases, which sometimes begin with convulsions. Unfortunately, there are many cases in which the cause of the convulsions is involved in obscurity and doubt, especially in rachitic children. In such cases we can only use our best judgment as to treatment.

